# Venous excess ultrasonography (VExUS) captures dynamic changes in volume status surrounding hemodialysis: A multicenter prospective observational study

**DOI:** 10.21203/rs.3.rs-4185584/v1

**Published:** 2024-04-08

**Authors:** Katarina Leyba, August Longino, Ryen Ormesher, Mary Krienke, Natalie Van Ochten, Katherine Zimmerman, Luke McCormack, Katharine Martin, Theresa Thai, Seth Furgeson, Isaac Teitelbaum, Joseph Burke, Ivor Douglas, Edward Gill

**Affiliations:** University of Colorado; University of Colorado; University of Colorado; University of Colorado; University of Colorado; University of Colorado; University of Colorado; University of Colorado School of Medicine; University of Colorado; Denver Health Medical Center; University of Colorado; University of Colorado; Denver Health Medical Center; University of Colorado

**Keywords:** End-stage renal disease (ESRD), hemodialysis (HD), point-of-care ultrasonography (POCUS), venous congestion, venous excess ultrasonography (VExUS), volume status

## Abstract

**Background:**

The evaluation of volume status is essential to clinical decision-making, yet multiple studies have shown that physical exam does not reliably estimate a patient’s intravascular volume. Venous excess ultrasound score (VExUS) is an emerging volume assessment tool that utilizes inferior vena cava (IVC) diameter and pulse-wave Doppler waveforms of the portal, hepatic and renal veins to evaluate venous congestion. A point-of-care ultrasound exam initially developed by Beaubein-Souligny et al., VExUS represents a reproducible, non-invasive and accurate means of assessing intravascular congestion. VExUS has recently been validated against RHC—the gold-standard of hemodynamic evaluation for volume assessment. While VExUS scores were shown to correlate with elevated cardiac filling pressures (i.e., right atrial pressure (RAP) and pulmonary capillary wedge pressure (PCWP)) at a static point in time, the ability of VExUS to capture dynamic changes in volume status has yet to be elucidated. We hypothesized that paired VExUS examinations performed before and after hemodialysis (HD) would reflect changes in venous congestion in a diverse patient population.

**Methods:**

Inpatients with end-stage renal disease undergoing intermittent HD were evaluated with transabdominal VExUS and lung ultrasonography before and following HD. Paired t-tests were conducted to assess differences between pre-HD and post-HD VExUS scores, B-line scores and dyspnea scores.

**Results:**

Fifty-six patients were screened for inclusion in this study. Ten were excluded due to insufficient image quality or incomplete exams, and forty-six patients (ninety-two paired ultrasound exams) were included in the final analysis. Paired t-test analysis of pre-HD and post-HD VExUS scores revealed a mean VExUS grade change of 0.82 (p<0.001) on a VExUS scale ranging from 0 to 4. The mean difference in B-line score following HD was 0.8 (p=0.001). There was no statistically significant difference in subjective dyspnea score (p=0.41).

**Conclusions:**

Large-volume fluid removal with HD was represented by changes in VExUS score, highlighting the utility of the VExUS exam to capture dynamic shifts in intravascular volume status. Future studies should evaluate change in VExUS grade with intravenous fluid or diuretic administration, with the ultimate goal of evaluating the capacity of a standardized bedside ultrasound protocol to guide inpatient volume optimization.

## BACKGROUND

The evaluation of volume status is essential to clinical decision-making in the intensive care unit, yet multiple studies have shown that physical exam does not reliably estimate a patient’s volume. Bedside assessments of intravascular volume remain subjective and often vary significantly between providers.^1–5^ Invasive methods (i.e., the direct measurement of central venous pressure via right heart catheterization (RHC)) remain the gold-standard for the evaluation of intravascular congestion, but these approaches carry risk, additional expense and are not universally available. More importantly, such procedures cannot be performed serially at the bedside to inform clinical decisions such as the initiation or discontinuation of volume resuscitation, diuresis or renal replacement therapy.^6,7^ Even indwelling pulmonary artery catheters, which can be utilized for serial monitoring over time, are associated with added cost and risk of complications.^8,9^ As we move towards the personalization of care for critically ill patients with conditions such as septic shock and decompensated heart failure, it is increasingly pressing to develop a standardized point-of-care method for evaluating volume status that can inform clinical decision-making in real-time.^10–17^

**Figure 1:**
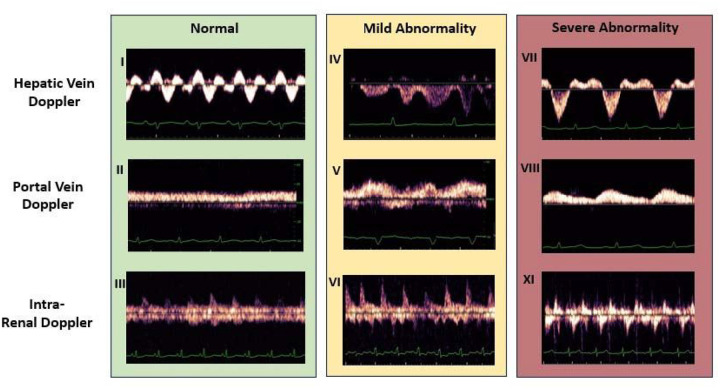
Representative pulse-wave Doppler waveforms for each VExUS component. Any patient whose IVC diameter is <2 cm is assigned a VExUS score of 0, indicating no venous congestion. For any patient who has an IVC diameter >2 cm, the scores of the component parts are totaled to contrive a composite score. Any combination of normal and mildly abnormal scores is assigned a VExUS score of 1, indicating mild venous congestion. Any patient with one severely abnormal waveform is assigned a VExUS score of 2, indicating moderate congestion. Two or more severely abnormal waveforms results in a VExUS grade of 3, indicating severe congestion.

Venous excess ultrasound score (VExUS) is an emerging volume assessment tool that utilizes inferior vena cava (IVC) diameter and pulse-wave Doppler waveforms of the portal, hepatic and renal veins to evaluate venous congestion (Fig. 1). A point-of-care ultrasound exam initially developed by Beaubein-Souligny et al., VExUS represents a reproducible, non-invasive and accurate means of assessing venous congestion.^18^ This four-point examination has been shown to surpass the limits of other bedside ultrasound exams (e.g., measurement of IVC diameter alone) and to correlate with clinically relevant outcomes, such as the development of acute kidney injury (AKI) in post-cardiac surgery patients and patients with acute coronary syndrome.^18–20^

More recently, VExUS has been validated against RHC.^20,21^ While VExUS scores were shown to correlate with elevated cardiac filling pressures (i.e., right atrial pressure (RAP) and pulmonary capillary wedge pressure (PCWP)) at a static point in time, the ability of VExUS to capture dynamic changes in volume status has yet to be elucidated. We hypothesized that paired VExUS examinations performed before and after hemodialysis (HD) would reflect changes in venous congestion in a diverse patient population.

## METHODS

### Enrollment and image acquisition

Inpatients scheduled to undergo intermittent HD at two tertiary medical centers near Denver, CO were screened for enrollment in this study from May to December 2023. Inclusion criteria were inpatient admission, age > 18 years, scheduled HD and ability to provide informed consent. Exclusion criteria included pregnancy, continuous renal replacement therapy, presence of an open abdominal wound, invasive mechanical ventilation, incarceration and inability to provide informed consent.

Enrolled patients were evaluated with two transabdominal VExUS and lung ultrasonography exams—one before and one after same-day HD. VExUS exams were performed using the standardized protocol previously described.^18,20^ In patients who had received a prior renal transplant, either of the native kidneys was scanned, but the transplanted kidney was not. Lung ultrasonography was performed using a standardized protocol that involved ultrasonographic examination of eight lung zones—four anterior zones, and two lateral zones on each side—to evaluate for the presence of B-lines (i.e., vertical sonographic lines that can indicate the presence of interstitial edema).^22^ A lung zone that contained > 3 B-lines was considered positive for B-lines.^23^ Sonographers recorded the number and distribution of lung zones with positive B-lines while performing both pre-HD and post-HD ultrasound exams. The sonographers were internal medicine resident physicians who had undergone remote and in-person training by physicians with experience performing the VExUS exam. Members of the research team were not part of the patients’ clinical team, and study data was not made available to enrolled patients or to clinical team members. Acquired images were anonymized and uploaded to a secure database.

### Image interpretation and data analysis

VExUS exams were graded by readers blinded to the patient’s clinical status. The interpretation team was comprised of internal medicine resident physicians who were proficient in the VExUS exam and had completed a 4-video imaging course on VExUS interpretation. The interpretation team did not participate in image acquisition. Images were interpreted and scored according to the standardized protocol previously described by Beaubien-Souligny et al.^18^ and utilized in the RHC validation study by Longino et al.^20^ Graders assigned a score to each component of the VExUS exam in addition to determining a composite score and an image quality score. Image quality was assessed using an ordinal scale from 1 (“uninterpretable”) to 5 (“unambiguously high quality”). Images with a quality score < 3 were excluded from the analysis. A sample of images was reviewed and interpreted by two graders to ensure consistency in image scoring.

Clinical characteristics of included patients were obtained via chart review and stored in a secure database. End-stage renal disease (ESRD) etiology was determined by reviewing clinical documentation from nephrology advanced practice providers, fellow physicians or attending physicians. Etiologies were not considered mutually exclusive, and more than one etiology was recorded for patients with multiple contributors to their renal disease. Left ventricular ejection fraction (LVEF) was determined from transthoracic echocardiogram (TTE) reports, considering only TTEs obtained within the past five years. If more than one TTE report was available, the LVEF value from the most recent report was used. Recent TTE reports and LVEF data were available for 93% of patients included in the final analysis.

Image graders were blinded to recorded clinical characteristics. Descriptive characteristics of the study cohort were summarized as mean ± standard deviation for continuous variable and frequency and percentage for categorical variables. The minimum IVC diameter (IVC_min_) (i.e., IVC diameter measured on inspiration) and maximum IVC diameter (IVC_max_) were measured as part of each VExUS exam and used to calculate IVC collapsibility indices (ICI) using the formula ICI = (IVC_max_ - IVC_min_)/IVC_max_.^24^ Paired t-tests were conducted to detect differences between pre-HD and post-HD VExUS scores (including both composite and component scores), B-line scores, patient-reported dyspnea scores, and pre-HD and post-HD IVC diameters and ICIs. VExUS grade was treated as a categorical variable throughout. Paired t-test analyses were also performed for prespecified subgroups (i.e., pre-HD VExUS score of 0 versus > 0, volume removal of < 1 L with HD versus volume removal of > 1 L with HD). All calculations were performed in R (version 4.3.2, R Foundation for Statistical Computing, Vienna, Austria) and RStudio (version 2023.12.1 + 402, RStudio, Inc., Boston, MA). A predetermined p-value of < 0.05 was considered statistically significant for all calculations.

## RESULTS

### Study population

Fifty-six patients were screened for inclusion in the study, and ten were excluded due to insufficient image quality or incomplete exams. Ninety-two paired VExUS exams were included in the final analysis. The mean age of all subjects was 60. Twenty-six (57%) were male, and twenty (43%) were female. The most common comorbidity was pulmonary hypertension (26%), followed by heart failure with reduced ejection fraction (22%). Patients with both AKI with unknown renal recovery and diagnosed ESRD were screened for inclusion in this study. All forty-six patients included in the final analysis carried a diagnosis of ESRD, and the most common ESRD etiology was diabetes mellitus (32.6%). The mean Charlson comorbidity index for all included patients was 5 (range 3–7) (Table 1). None of the patients enrolled in this study were undergoing positive pressure ventilation.

### Paired pre-HD and post-HD VExUS scores

**Figure 2:**
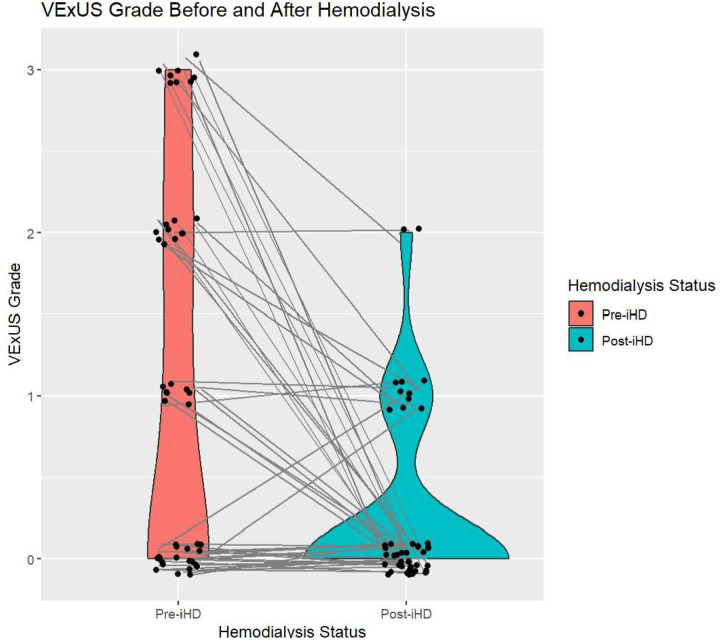
VExUS grades before and after HD. Matched pre-HD and post-HD VExUS grades. Paired t-test analysis of pre-HD and post-HD VExUS scores revealed a mean VExUS grade change of 0.82 (p<0.001).

The volume of fluid removed with HD ranged from zero to four liters, with a mean fluid removal of 1.9 L. The mean VExUS score before HD was 1.1, and the mean VExUS score after HD was 0.28 (Fig. 2). Nearly half of patients (46% or n = 21) had a VExUS grade of 0 prior to HD, indicating an absence of venous congestion. A smaller portion of patients demonstrated mild or moderate congestion, with 17% having a pre-HD VExUS grade of 1 and 20% having a pre-HD VExUS grade of 2. Only 17% of patients had severe venous congestion (as indicated by a VExUS grade of 3) before HD. After HD, 76% of patients had a VExUS grade of 0, 20% had a VExUS grade of 1 and 4.3% had a VExUS grade of 2. There were no patients with a VExUS grade of 3 (i.e., severe venous congestion) following HD (Table 2).

Paired t-test analysis of pre-HD and post-HD VExUS scores revealed a statistically significant difference in VExUS grade before and after HD, with a mean VExUS grade change of 0.82 (p < 0.001). While there were significant differences in IVC diameter before and after HD, there was not a significant change in ICI. The mean pre-HD IVC_min_ was 1.46 cm compared to a mean post-HD IVC_min_ of 1.27 cm (p = 0.01), and the mean pre-HD IVC_max_ was 2.01 cm compared to a mean post-HD IVC_max_ of 1.88 cm (p = 0.048). ICI was similar pre-HD and post-HD (0.30 and 0.33, respectively, with p = 0.21). The individual components of hepatic vein VExUS score and portal vein VExUS score signicantly decreased after HD, with mean pre-HD scores of 0.87 and 0.87 for both components, and mean post-HD scores of 0.37 (p < 0.001) and 0.3 (p < 0.001), respectively. Interestingly, there was not a statistically significant decrease in renal vasculature VExUS score before and after HD. Rather, the mean renal vasculature score increased from 0.33 pre-HD to 0.83 post-HD (p = 0.01). Pre-HD and post-HD component and composite VExUS scores are further outlined in Table 2. The mean difference in B-line score following HD was 0.8 (p = 0.001). There was no statistically significant difference in subjective dyspnea score before and after HD (p = 0.41).

For patients who had a pre-HD VExUS score > 0 (indicating some degree of venous congestion), the mean VExUS grade change was 1.56 (p < 0.001), and for patients with a pre-HD VExUS score of 0 (indicating an absence of venous congestion), there was no statistically significant change in VExUS score following HD (p = 0.16). Similarly, the delta VExUS score (i.e., pre-HD VExUS score minus post-HD VExUS score) was higher in patients who had a greater volume of fluid removed with HD. For patients who had > 1 L of volume removed during HD, the mean delta VExUS was 1.12 (p < 0.001), yet there was no statistically significant change in VExUS grade in patients who had < 1 L removed with HD (p = 0.07).

## DISCUSSION

Large-volume fluid removal with HD was represented by changes in VExUS score, highlighting the utility of the VExUS exam to capture dynamic shifts in volume status. This multicenter study is the first interrogation of the ability of VExUS to appraise rapid changes in a patient’s hemodynamic state, particularly venous congestion.

Importantly, the VExUS exam has previously been shown to outperform both ICI and the measurement of IVC diameter—two metrics routinely incorporated into standard echocardiogram protocols—in the estimation of RAP. Our group recently published a study validating VExUS against RHC, an analysis that found that the area under the curve (AUC) for VExUS grade as a predictor of RAP > 10 mmHg was 0.9, whereas the AUC for ICI and IVC diameter as predictors of RAP > 10 mmHg were 0.65 and 0.77, respectively. Similarly, the AUC for VExUS grade as predictor of RAP < 7 mmHg was 0.79, while the AUC for ICI and IVC diameter as predictors of RAP < 7 mmHg were 0.62 and 0.74, respectively, highlighting the fact that VExUS was superior to ICI or IVC diameter for the detection of euvolemia or hypovolemia in addition to venous congestion. These findings hold true in the current study as well. Whereas the VExUS exam was able to detect significant changes in volume status, ICI was not. There was no statistically significant change in paired ICI scores before and after HD, underscoring the limitations of this unidimensional metric.^25,26^ In fact, the American Society of Echocardiography’s (ASE) guidelines for the estimation of RAP via ICI (i.e., an ICI < 50% is indicative of an RAP 10–20 mmHg) would suggest that both the pre-HD and post-HD cohort trended towards hypervolemia given their ICIs of 0.30 and 0.33, respectively.^27^ This interpretation does not reflect the fact that a mean volume change of nearly negative 2 L occurred between paired pre-HD and post-HD ultrasound exams. While there was a statistically significant difference in pre-HD and post-HD IVC diameter, an interpretation of IVC diameter utilizing ASE guidelines would yield a similarly static conclusion. In the current analysis, the mean pre-HD IVC_max_ was 2.01 cm, and the mean post-HD IVC_max_ was 1.88 cm. According to ASE criteria, an IVC diameter of < 2.1 cm is suggestive of a RAP < 5 mmHg.^27^ Utilizing this schema, both the pre-HD and post-HD cohorts would have been considered to be uncongested, but—similar to ICI—there would have been no indication that a significant volume loss occurred between paired pre-HD and post-HD exams, as an IVC diameter of 2.01 cm and an IVC diameter of 1.88 cm both signal a RAP < 5 mmHg.

Overall, these findings underscore the constraints of binary metrics (e.g., IVC size > 2.1 cm versus < 2.1cm or ICI > 50% versus < 50%) for the estimation of a patient’s degree of venous congestion and highlight an opportunity to expand on the IVC evaluation that is integrated into current echocardiographic protocols. VExUS represents an objective, validated, multidimensional, and more nuanced appraisal of hemodynamic status that moves beyond the assessment of IVC alone to incorporate data from multiple encapsulated abdominal organs in addition to the IVC. The tiered scoring system utilized by VExUS (i.e., scores 0–3, representing no venous congestion, mild congestion, moderate congestion and severe congestion, respectively) expands on current schema for the estimation of venous hypertension and allows for more gradation in the valuation of a patient’s volume status. The supplement of three additional views (i.e., the hepatic vein, portal vein and intralobar renal vasculature) with pulse-wave Doppler wave forms has the potential to augment the already marked clinical utility of volume assessment via ultrasound exam, either as part of full echocardiographic exam or a stand-alone limited protocol.

Beyond the integration of VExUS into formal sonographic procedures, there also lies an opportunity to incorporate VExUS into routine clinical care as an easily accessible, point-of-care exam. VExUS has the unique advantage of being a non-invasive, inexpensive and reproducible ultrasound protocol, making it highly accessible to bedside clinicians. As such, the VExUS exam can be used to serially to assess changes in a patient’s volume status over time, marking it as an ideal tool for evaluating rapid changes in a patient’s hemodynamic status in real-time, such as diuresis in hypervolemic states (e.g., decompensated heart failure) or volume resuscitation in rapidly evolving clinical scenarios such as sepsis. Future studies should evaluate change in VExUS grade with intravenous fluid or diuretic administration in various patient populations, with the ultimate goal of evaluating the capacity of a standardized bedside ultrasound protocol to guide inpatient volume optimization.

While this analysis demonstrates the ability of the VExUS exam to capture changes in venous congestion that occur in the setting of large-volume fluid removal in patients undergoing HD, it does not assess the ability of the VExUS exam to detect volume loss in other clinical settings (e.g., diuresis, gastrointestinal losses or hemorrhage), nor does it assess the ability of the VExUS exam to detect rapid volume expansion. Moreover, this study included only patients with ESRD, and the effect of chronic renal disease on the renal component of the VExUS exam has not been directly investigated, leaving open the possibility that ESRD necessitating HD may impair the interpretation of renal vasculature pulse-wave Doppler waveforms. It is important to note, however, that the VExUS exam was validated in diverse patient populations with multiple compounding comorbidities and is intended as a tool to interrogate venous congestion across a spectrum of critical illness.^20,21^ Beaubien-Souligny et al.’s seminal study, for instance, investigated the ability of VExUS to predict AKI in patients who had recently undergone cardiac surgery with the use of cardiopulmonary bypass, and Longino et al.’s recent analyses interrogating the utility of VExUS to estimate RAP occurred in patients undergoing RHC, overall suggesting that the multivariate nature of the exam confers robustness and validity even in the setting of chronic disease.^18,20,21^ In the current analysis, the renal vasculature score did not decrease between paired pre-HD and post-HD exams, but the composite VExUS score decreased concordantly with the remaining component scores, suggesting that the redundancy of the overall VExUS exam is effective at mitigating variation between patients and disease states. Future studies are warranted to expressly validate VExUS in specific subpopulations, including patients with ESRD.

## CONCLUSIONS

Large-volume fluid removal with HD was represented by changes in VExUS score, highlighting the utility of the VExUS exam to capture dynamic shifts in volume status. VExUS holds potential as an addition to formal sonographic protocols and as a point-of-care bedside tool that can be utilized to detect changes in venous congestion in real-time. Future studies should evaluate change in VExUS grade with intravenous fluid or diuretic administration in various patient populations, with the ultimate goal of evaluating the capacity of a standardized bedside ultrasound protocol to guide inpatient volume optimization.

## Data Availability

All original ultrasound images and datasets are available upon request to the corresponding author.

## Abbreviations

AKI: acute kidney injury
ASE: American Society of Echocardiography
AUC: area under the curve
ESRD: end-stage renal disease
HD: hemodialysis
IVC: inferior vena cava
ICI: IVC collapsibility index
LVEF: left ventricular ejection fraction
PCWP: pulmonary capillary wedge pressure
RAP: right atrial pressure
RHC: right heart catheterization
TTE: transthoracic echocardiogram
VExUS: venous excess ultrasounds score
